# Development of an artificial intelligent model for pre-endoscopic screening of precancerous lesions in gastric cancer

**DOI:** 10.1186/s13020-024-00963-5

**Published:** 2024-06-29

**Authors:** Lan Wang, Qian Zhang, Peng Zhang, Bowen Wu, Jun Chen, Jiamin Gong, Kaiqiang Tang, Shiyu Du, Shao Li

**Affiliations:** 1 Institute for TCM-X, MOE Key Laboratory of Bioinformatics, Bioinformatics Division, BNRIST, Department of Automation, Tsinghua University, Beijing, China; 2 Department of Epidemiology and Health Statistics, School of Public Health, Fujian Medical University, Fuzhou, China; 3 Department of Control Science and Intelligence Engineering, Nanjing University, Nanjing, China; 4 Department of Gastroenterology, China-Japan Friendship Hospital, Chaoyang District, Beijing, China

**Keywords:** Deep learning, Tongue images, Inquiry information, Precancerous lesions of gastric cancer, Pre-endoscopic screening

## Abstract

**Background:**

Given the high cost of endoscopy in gastric cancer (GC) screening, there is an urgent need to explore cost-effective methods for the large-scale prediction of precancerous lesions of gastric cancer (PLGC). We aim to construct a hierarchical artificial intelligence-based multimodal non-invasive method for pre-endoscopic risk screening, to provide tailored recommendations for endoscopy.

**Methods:**

From December 2022 to December 2023, a large-scale screening study was conducted in Fujian, China. Based on traditional Chinese medicine theory, we simultaneously collected tongue images and inquiry information from 1034 participants, considering the potential of these data for PLGC screening. Then, we introduced inquiry information for the first time, forming a multimodality artificial intelligence model to integrate tongue images and inquiry information for pre-endoscopic screening. Moreover, we validated this approach in another independent external validation cohort, comprising 143 participants from the China-Japan Friendship Hospital.

**Results:**

A multimodality artificial intelligence-assisted pre-endoscopic screening model based on tongue images and inquiry information (AITonguequiry) was constructed, adopting a hierarchical prediction strategy, achieving tailored endoscopic recommendations. Validation analysis revealed that the area under the curve (AUC) values of AITonguequiry were 0.74 for overall PLGC (95% confidence interval (CI) 0.71–0.76, p < 0.05) and 0.82 for high-risk PLGC (95% CI 0.82–0.83, p < 0.05), which were significantly and robustly better than those of the independent use of either tongue images or inquiry information alone. In addition, AITonguequiry has superior performance compared to existing PLGC screening methodologies, with the AUC value enhancing 45% in terms of PLGC screening (0.74 vs. 0.51, p < 0.05) and 52% in terms of high-risk PLGC screening (0.82 vs. 0.54, p < 0.05). In the independent external verification, the AUC values were 0.69 for PLGC and 0.76 for high-risk PLGC.

**Conclusion:**

Our AITonguequiry artificial intelligence model, for the first time, incorporates inquiry information and tongue images, leading to a higher precision and finer-grained pre-endoscopic screening of PLGC. This enhances patient screening efficiency and alleviates patient burden.

## Introduction

According to recent surveys, gastric cancer is the fourth leading cause of cancer-related deaths worldwide and the second in China [1]. There are approximately 480,000 new cases and 370,000 deaths of gastric cancer in China each year, accounting for half of the cases in the world [2]. Gastric cancer is thought to develop from precancerous lesions of gastric cancer (PLGC) (e.g., chronic atrophic gastritis, intestinal metaplasia, or gastric epithelial dysplasia), and the graded screening and diagnosis of natural populations for PLGC is essential to reduce gastric cancer mortality [3–5]. However, the screening and diagnosis of gastric diseases still rely on gastroscopy, but its application is greatly limited because of its invasiveness, high cost, and the need for professional endoscopists [6]. Meanwhile, endoscopic screening is not suitable for large-scale natural populations, especially in rural China. Alternatively, the application of serum markers that are commonly used as screening factors in various gastric cancer risk assessment methods, such as pepsinogen I/II and gastrin-17, has been limited for risk screening in natural populations due to its invasiveness, and the high sensitivity and specificity thresholds [7, 8]. Therefore, there is an urgent need for affordable and non-invasive screening methods suitable for large-scale natural populations to improve diagnostic efficiency and reduce the incidence of gastric cancer.

Early studies have indicated that non-invasive features, including imaging characteristics and clinical phenotypic information, have the potential to predict the occurrence and progression of PLGC. The theory of Traditional Chinese medicine (TCM) suggests that the tongue's shape, color, size, and coating characteristics detected using tongue images reflect health status and disease severity/progression, and recent studies have shown the potential for tongue surface and color characteristics to assist in the diagnosis of PLGC [9, 10]. For digestive diseases, tongue image characteristics have been found to correlate with gastroscopic observations and predict gastric mucosal health [11, 12]. Additionally, interrogating inquiry information (e.g., living habits, dietary preferences, and physical symptoms) is crucial in understanding the disease and medical history [13]. In this regard, recent studies have built risk prediction models for PLGC before endoscopy using demographics and clinical risk factors, including helicobacter pylori (Hp) infection, sex, age, race/ethnicity, and smoking status [14–16]. However, the integration of tongue images and inquiry information for high-precision endoscopic screening of PLGC to facilitate precise endoscopic recommendations has not been studied.

With the rapid development of artificial intelligence (AI) technology, machine learning algorithms based on deep neural networks can accurately analyze diagnostic clinical images, identify therapeutic targets, and process large datasets, which can play a role in screening and diagnosis of a variety of diseases [17–21]. Pan et al. reviewed studies related to AI methods for lung cancer risk prediction, diagnosis, prognosis, and treatment response monitoring [22]. Wang et al. constructed an AI-based model of two-dimensional shear wave elastography of the liver and spleen to precisely assess the risk of GEV and high-risk gastroesophageal varices [23]. Ma et al. constructed the deep learning model for screening precancerous lesions of gastric cancer based on tongue images [24]. Li et al. found that both tongue images and the tongue-coating microbiome can be used as tools for the diagnosis of gastric cancer [25]. However, the value of these features in pre-endoscopic PLGC risk screening remains uncertain, and integrating these features based on AI to achieve a more refined PLGC risk screening still poses significant challenges.

In this study, we integrated tongue image data and inquiry data to construct an AI-based multimodal model to assist in pre-endoscopic screening of PLGC. We evaluated the performance of predicting PLGC and high-risk PLGC in a cohort of patients diagnosed with chronic gastritis in Fujian, China, and assessed the superiority of multimodal fusion over single modality. Additionally, we validated the model's performance in another independent cohort.

## Materials and methods

### Design and overview

This research recruited a cohort of patients diagnosed with chronic gastritis from Fujian, China, and the recruitment period spanned from December 2022 to December 2023. Those who volunteered to participate were included in this study. This study was approved by the ethics committee of the Fujian Medical University (Approval number 58 in 2020). Fujian is one of the regions with a high incidence of gastric cancer in China, and the local people have an urgent need for screening for gastric disease symptoms such as PLGC. Therefore, we selected patients in this region as the study subjects [26–28].

### Patient enrollment

One thousand and thirty-four potentially eligible patients were enrolled in this study. The inclusion criteria were as follows: (1) age between 18 and 70 years; (2) having the gastroscopy examination results saved within the past three months, or will undergo gastroscopy examination in the coming three months; (3) no previous diagnosis of cancer; (4) residing locally and being willing to cooperate with doctor's follow-up; and (5) providing written informed consent. Patients were excluded for the following reasons: (1) cancer patients; (2) contraindications for endoscopic examination; (3) pregnant women, women planning pregnancy, as well as lactating women; (4) cardiovascular, pulmonary, renal, endocrine, neurological, and hematological disorders; (5) mental disorder; and (6) unwilling to participate or poor compliance.

### Tongue images and inquiry information

It is recommended that patients adhere to a standardized procedure to acquire high-quality tongue images. Patients are advised to present themselves in natural light conditions during the morning, ensuring an empty stomach. Patients should protrude their tongue from the oral cavity, with particular attention to positioning the tip slightly downward and flattening the surface to ensure the entire tongue body is adequately visualized.

Simultaneously, the healthcare practitioner will engage in a comprehensive traditional Chinese medicine consultation with patients. This consultation encompasses an exploration of demographic details, such as gender, along with an assessment of pertinent lifestyle factors, including a history of smoking and alcohol consumption. Additionally, an inquiry into the patient's family medical history, dietary habits, and an evaluation of physical symptoms will be conducted. The physical symptoms evaluation involves an assessment of potential discomfort in the stomach and mouth, the patient's mental state, and their bowel and urinary habits.

### Endoscopic evaluation

Two independent gastroenterology experts, each of whom had carried out more than 1000 endoscopies, performed esophagogastroduodenoscopy (EGD) on all patients. The biopsy results were reported as normal, superficial gastritis, chronic atrophic gastritis, intestinal metaplasia, or intraepithelial neoplasia, and a diagnosis was assigned to each participant based on the most severe histological finding in the biopsy. The Hp infection status was determined by enzyme-linked immunosorbent assay of plasma IgG. The procedure was conducted up to 3 months before or after the acquisition of images and traditional Chinese medicine inquiry, and the operators were unaware of the results of the tongue examination and inquiry information.

### Single-modality deep learning risk prediction models

#### Tongue images deep learning risk prediction (single-tongue) model

This section proposes Single-Tongue, a new diagnostic approach based on single-modality deep learning using tongue images to predict PLGC and high-risk PLGC. We applied the Segment Anything Models [29] to segment tongue images to extract the features of the effective area and avoid the influence of irrelevant edge noise information.

All patients were randomly divided into training and validation cohorts. The training cohort was utilized to train a deep neural network designed for this study. The performance of the trained model was evaluated through its application to the validation cohort. In order to extract the features from the tongue images, we employed a pre-trained ResNet framework that had been previously trained on the ImageNet dataset [30]. Distinct from convolutional neural networks (CNNs), ResNet tackles the issues of vanishing gradients and network degradation by introducing direct skip connections within the network, which retain a certain proportion of the output from the previous network layer. Data augmentation techniques such as random cropping, flipping, and rotation were applied to all image data to mitigate overfitting. The images were passed through ResNet during training, specifically through the bottleneck and residual units. In our study, the extraction of tongue image features by our model is achieved through an automated learning process rather than manual selection or definition by us. The model automatically extracts features such as tongue coating color, thickness, moisture, and tongue body shape through convolutional pooling layers. After passing through 12 bottleneck layers, an adaptive average pooling operator was used to obtain image features, which were then flattened to a size of 2048*1. The final classification results were generated through a softmax layer. In the single-modality experiment, two binary classification networks were trained to determine the presence or absence of PLGC or high-risk PLGC.

#### Inquiry information deep learning risk prediction (single-inquiry) model

The inquiry information encompasses variables such as sex, age, and the individuals' history of smoking and alcohol consumption. Furthermore, it incorporates the family medical history, dietary habits, and physical symptoms of the patients, including discomfort in the stomach and mouth and their mental state. We employed a segregation approach by categorizing the features into numerical and factor types to enhance the effectiveness of utilizing the inquiry information. The numerical features were subjected to min–max normalization, scaling them between 0 and 1. On the other hand, the factor features were transformed into numeric vectors using keyword-based encoding techniques.

After feature filtering and mapping, the inquiry information was input into a multilayer perceptron to obtain corresponding feature vectors. Then, two binary classification networks were trained to determine the presence or absence of PLGC or high-risk PLGC.

### Multimodality deep learning risk prediction (AITonguequiry) model

Medical data are frequently multimodal. For instance, both tongue images and inquiry information encompass details associated with PLGC. Consequently, in this section, we integrated these two modalities with an attentional mechanism.

In the multimodality experiment, similar to the single-modality experiment, we trained two binary classification networks to determine the presence or absence of PLGC or high-risk PLGC. We employed the dropout method to eliminate a certain proportion of model parameters. Subsequently, we utilized the feature embedding method to align the feature vectors of tongue images and inquiry information for comprehensive patient information utilization.

### Statistical analysis

The prediction results were validated by quantitative indexes, including sensitivity, specificity, positive predictive value and negative predictive value. The chi-squared test and t test were used to determine whether there was any significant difference in patient characteristics. The area under the receiver operating characteristic (ROC) curve (AUC) was used to estimate the probability that the model would produce a correct prediction. The DeLong test was used to test whether there was a significant difference in risk prediction between AITonguequiry and other methods.

## Results

### Patient characteristics

In this research, a cohort of 1034 participants was recruited in Fujian, China, and the recruitment period spanned from December 2022 to December 2023. Among these patients, NPLGC (Non-precancerous lesions of gastric cancer) was documented in 855 (82.61%) patients, and PLGC was documented in 180 (17.39%) patients. Among PLGC, low-risk PLGC and high-risk PLGC account for 346 (65.90%) and 179 (34.10%), respectively. After randomization of these patients, 828 patients were assigned to the training cohort. The other 207 patients composed the validation cohort.

The baseline characteristics of the study population are summarized in Tables 1 and 2. Overall, the average age of patients with PLGC was 62, and the standard deviation was 7. In terms of gender, the proportion of males with PLGC (180[34.29%]), and females with PLGC (345[65.71%]). Between the training and validation cohorts, there were no significant differences in any of the baseline characteristics (p > 0.05) or in the distribution of patients between NPLGC and PLGC.

**Table 1:** Baseline characteristics of the study cohort: NPLGC and PLGC

Characteristic	Training and validation dataset^a^		p-value^b^
	NPLGC, N = 509	PLGC, N = 525	
Age (years)	59 (9)	61 (8)	< 0.001
Sex (female/male)	343/166	345/180	0.57
Family history (yes/no)	39/470	47/478	0.45
Drinking (yes/no)	136/373	144/381	0.80
Smoking (yes/no)	141/368	153/372	0.61
Hp (positive/negative/unknown)	14/31/464	19/32/474	0.73

*PLGC* precancerous lesions of gastric cancer, *NPLGC* non-precancerous lesions of gastric cancer, *Hp* helicobacter pylori

^a^Mean (SD); n (%)

^b^Welch Two Sample t-test; Pearson’s Chi-squared test; Fisher's exact test

**Table 2:** Baseline characteristics of the study cohort: low-risk PLGC and high-risk PLGC

Characteristic	Training and validation dataset^a^		p-value^b^
	Low-risk PLGC, N = 346	High-risk PLGC, N = 179	
Age (years)	61 (8)	62 (7)	0.45
Sex (female/male)	240/106	105/74	0.014
Family history (yes/no)	27/319	20/159	0.20
Drinking (yes/no)	77/269	67/112	< 0.001
Smoking (yes/no)	86/260	67/112	0.003
Hp (positive/negative/unknown)	6/19/321	13/13/153	0.004

*PLGC* precancerous lesions of gastric cancer, *NPLGC* non-precancerous lesions of gastric cancer, *Hp* helicobacter pylori

^a^Mean (SD); n (%)

^b^Welch Two Sample t-test; Pearson's Chi-squared test; Fisher's exact test

### Construction of the AITonguequiry model

**Fig. 1:**
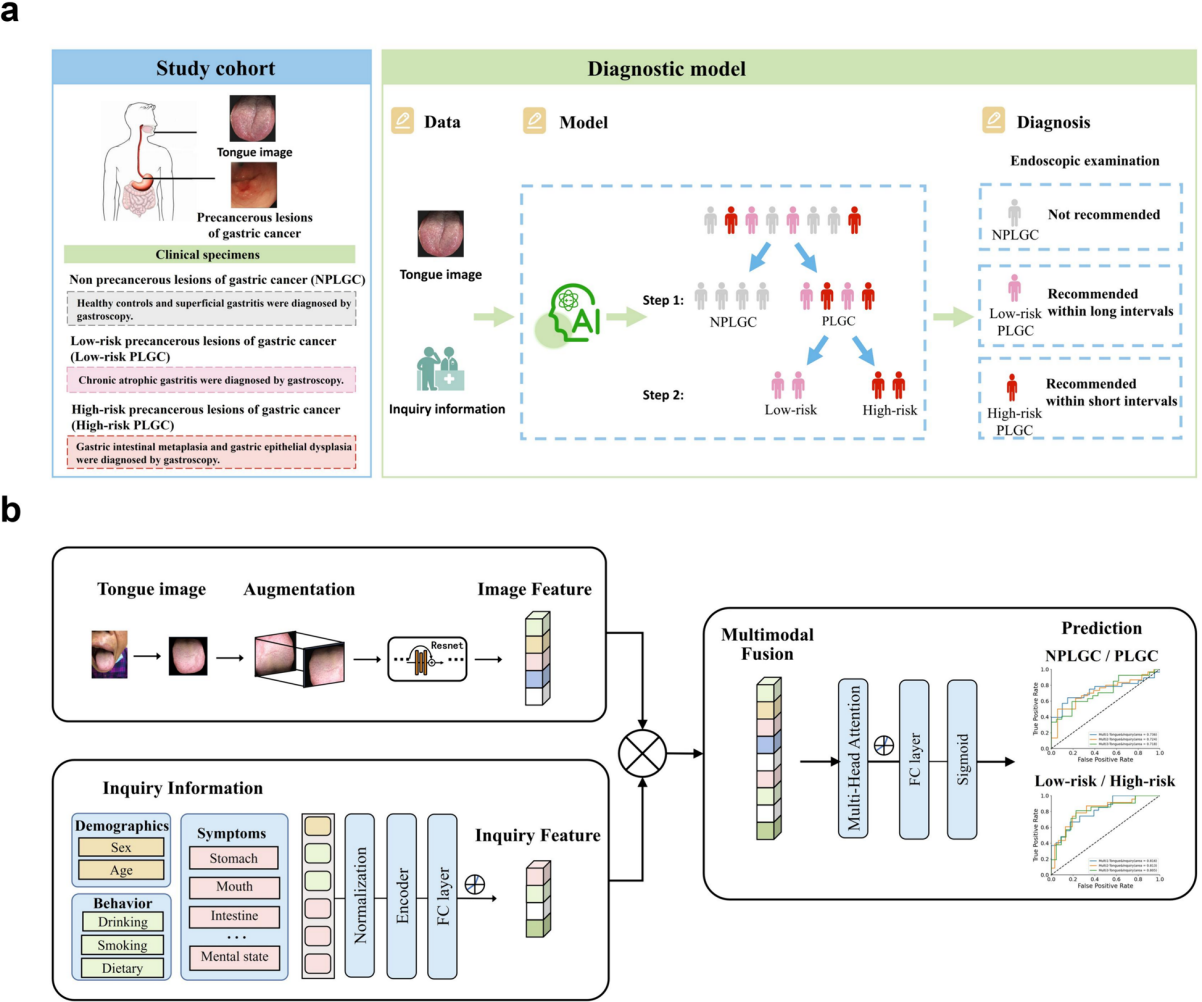
AITonguequiry flow chart. **a** The study cohort and the diagnostic model. **b** A deep learning–based multimodality classification model was developed to assess the risk of PLGC and high-risk PLGC based on the tongue images and inquiry information. *PLGC* precancerous lesions of gastric cancer, *NPLGC* non-precancerous lesions of gastric cancer

We build a deep learning risk prediction model based on tongue images and inquiry information (AITonguequiry) from 1034 patients to evaluate their potential in the grading screening and diagnosis of PLGC. The AITonguequiry flow chart is shown in Fig. 1. As shown in Fig. 1a, the study cohort and the diagnostic model were designed to assess the risk of PLGC and high-risk PLGC based on tongue images and inquiry information. The detailed multimodality model is shown in Fig. 1b. The patients were categorized into two groups: NPLGC and PLGC, and the PLGC group was further divided into low-risk PLGC (chronic atrophic gastritis) and high-risk PLGC (intestinal metaplasia or gastric epithelial dysplasia) stages. We advocate for patients predicted with PLGC to undergo endoscopic examination, with a concurrently recommending prompt endoscopic examination for those predicted with high-risk PLGC.

### Comparison of the AITonguequiry model, single modality models and baseline characteristics

We chose Single-Tongue, Single-Inquiry and baseline characteristics to identify the presence or absence of PLGC and high-risk PLGC.

The selection of baseline characteristics is based on individuals aged ≥ 45 who meet any of the following criteria, which are indicative of a high-risk profile for gastric cancer: (1) Long-term residence in high-incidence areas of gastric cancer; (2) Hp infection; (3) History of chronic atrophic gastritis, gastric ulcer, gastric polyp, residual stomach after surgery, hypertrophic gastritis, pernicious anemia, or other precancerous diseases of the stomach; (4) First-degree relatives with a history of gastric cancer; (5) Presence of other high-risk factors for gastric cancer such as high salt intake, pickled diet, smoking, and heavy alcohol consumption [31–34]. Since our screening is conducted in high-risk areas, we ignore the first criterion.

**Fig. 2:**
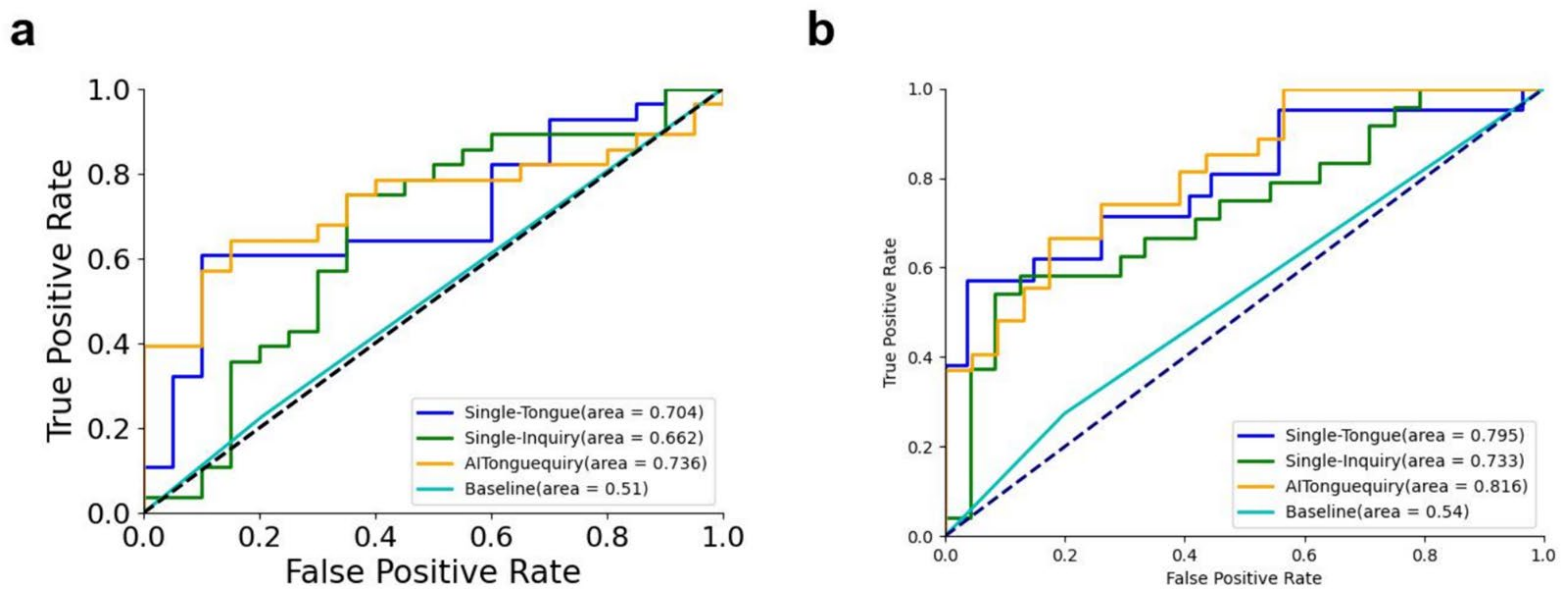
Comparison of ROC curves between different methods for classifying the presence or absence of PLGC and high-risk PLGC in the validation cohorts. **a** Presence or absence of NPLGC/PLGC in the validation cohorts. **b** Presence or absence of low-risk PLGC/high-risk PLGC in validation cohorts. *PLGC* precancerous lesions of gastric cancer, *NPLGC* Non-precancerous lesions of gastric cancer, *Single-Tongue* single-modality deep learning risk prediction with tongue images, *Single-Inquiry* single-modality deep learning risk prediction with inquiry information, *AITonguequiry* multimodality deep learning risk prediction with tongue images and inquiry information

In identifying the presence or absence of PLGC, AITonguequiry demonstrated statistically higher AUCs than Single-Tongue, Single-Inquiry and baseline characteristics (p < 0.05) (Fig. 2a; Table 3). Impressively, AITonguequiry had an AUC of 0.736 for the diagnosis of PLGC, which was higher than other methods (Fig. 2a, all p < 0.05). The sensitivity and specificity analyses also demonstrated that AITonguequiry universally outperformed the Single-Tongue, Single-Inquiry and baseline characteristics for assessing PLGC and high-risk PLGC (Table 3).

**Table 3:** Diagnostic performance of AITonguequiry for the assessment of NPLGC/PLGC and low-risk PLGC/high-risk PLGC in the validation cohorts

	AUC	Specificity (%)	Sensitivity (%)	PPV (%)	NPV (%)
Step 1: NPLGC/PLGC					
Single-Tongue	0.70* (0.68–0.73)	54.55 (53.14–56.02)	86.67 (84.23–89.18)	46.43 (43.65–49.31)	90.00 (87.99–91.96)
Single-Inquiry	0.66* (0.64–0.69)	62.50 (59.63–65.58)	68.75 (67.13–70.32)	78.57 (76.33–80.96)	50.00 (46.58–53.42)
AITonguequiry	0.74 (0.71–0.76)	66.67 (63.94–69.43)	73.33 (71.59–75.04)	78.57 (76.16–80.87)	60.00 (56.66–63.27)
Step 2: Low-risk PLGC/high-risk PLGC					
Single-Tongue	0.79* (0.78–0.82)	90.00 (86.97–92.58)	52.63 (51.54–53.69)	95.24 (93.60–96.57)	33.33 (30.58–36.00)
Single-Inquiry	0.73* (0.71–0.75)	64.00 (61.91–66.18)	65.22 (63.03–67.39)	62.50 (59.50–65.60)	66.67 (63.80–69.50)
AITonguequiry	0.82 (0.80–0.83)	66.67 (64.61–68.66)	78.26 (76.09–80.44)	66.67 (63.79–69.44)	78.26 (75.52–80.85)

The AUC of AITonguequiry was statistically compared with the AUCs of Single-Tongue and Single-Inquiry (*P < 0.05; **P < 0.01)

*AUC* area under the receiver operating characteristic curve, *PLGC* precancerous lesions of gastric cancer, *NPLGC* Non-precancerous lesions of gastric cancer, *Single-Tongue* single-modality deep learning risk prediction with tongue images, *Single-Inquiry* single-modality deep learning risk prediction with inquiry information, *AITonguequiry* multimodality deep learning risk prediction with tongue images and inquiry information, *PPV* positive predictive value, *NPV* negative predictive value

In identifying the presence or absence of high-risk PLGC, AITonguequiry demonstrated statistically higher AUCs than Single-Tongue, Single-Inquiry and baseline characteristics (p < 0.05) (Fig. 2b; Table 3). Impressively, AITonguequiry had an AUC of 0.816 for the diagnosis of high-risk PLGC, which was higher than other methods (Fig. 2b, all p < 0.05). The sensitivity and specificity analyses also demonstrated that AITonguequiry universally outperformed the Single-Tongue, Single-Inquiry and baseline characteristics for assessing PLGC and high-risk PLGC (Table 3).

### Evaluation of the diagnostic robustness of the AITonguequiry model

**Fig. 3:**
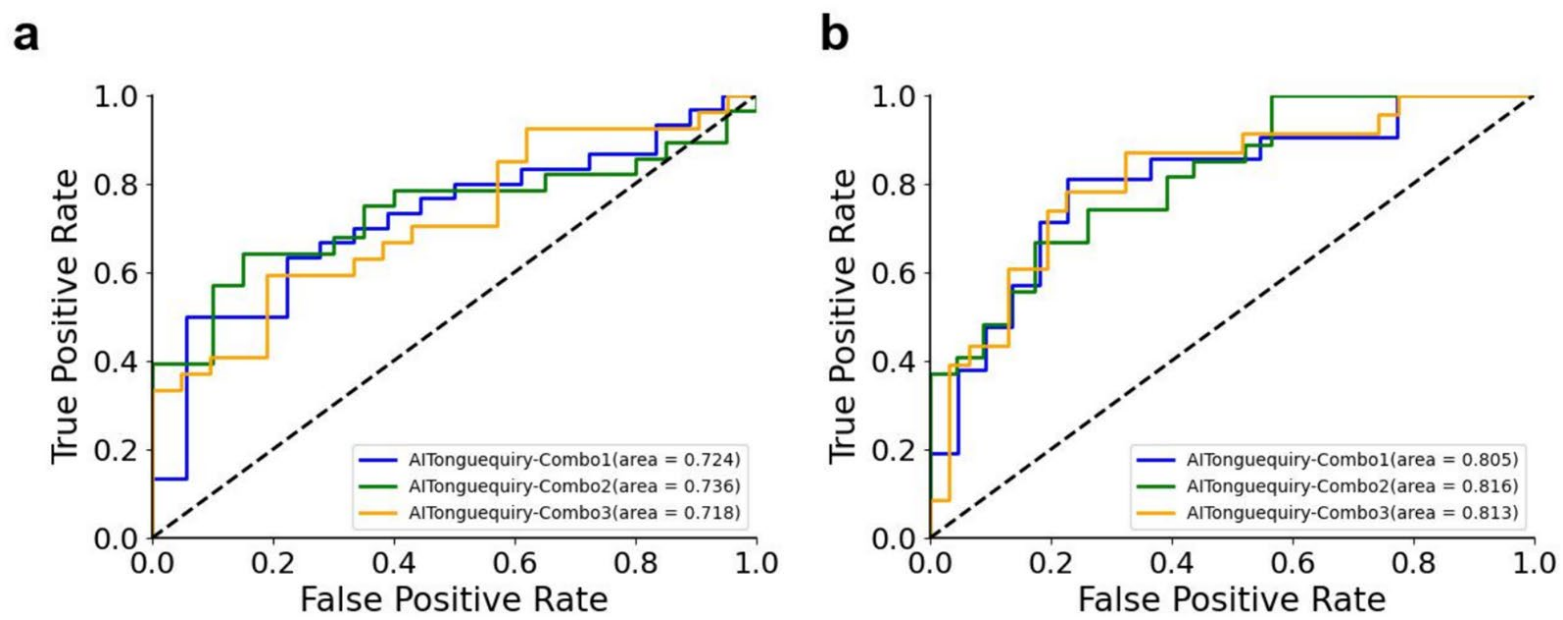
Comparison of receiver operating characteristic (ROC) curves among different combinations using AITonguequiry. **a** Presence or absence of NPLGC/PLGC in the validation cohorts. **b** Presence or absence of low-risk PLGC/high-risk PLGC in validation cohorts. *PLGC* precancerous lesions of gastric cancer, *NPLGC* Non-precancerous lesions of gastric cancer, *AITonguequiry* multimodality deep learning risk prediction with tongue images and inquiry information

All patients were randomly divided into training and validation cohorts. Simultaneously, all the images of each patient were allocated to the cohort corresponding to that patient, ensuring that there was no simultaneous presence of different images from the same patient in both cohorts. In either the validation cohort, the results in the three ROC curves always overlapped each other (Fig. 3), and no significant differences were found (all p > 0.05, Table 4). These results revealed that AITonguequiry showed consistent and robust performance regardless of the data's origin from different medical centers, as long as the number of enrolled patients in different training cohorts remained fairly constant.

**Table 4:** Diagnostic robustness of AITonguequiry for the assessment of NPLGC/PLGC and low-risk PLGC/high-risk PLGC in the validation cohorts

	AUC	Specificity (%)	Sensitivity (%)	PPV (%)	NPV (%)
Step 1: NPLGC/PLGC					
Combo 1	0.74 (0.71–0.76)	66.67 (63.91–69.44)	73.33 (71.62–75.06)	78.57 (76.16–80.88)	60.00 (56.90–63.27)
Combo 2	0.72 (0.70–0.75)	54.55 (52.21–56.88)	76.92 (75.00–78.79)	66.67 (64.00–69.20)	66.67 (63.33–70.00)
Combo 3	0.72 (0.69–0.74)	60.00 (57.32–62.52)	67.86 (65.96–69.73)	70.37 (67.73–73.07)	57.14 (53.83–60.46)
Step 2: Low-risk PLGC/high-risk PLGC					
Combo 1	0.82 (0.80–0.83)	66.67 (64.63–68.66)	78.26 (75.95–80.51)	66.67 (63.80–69.35)	78.26 (75.30–80.96)
Combo 2	0.81 (0.79–0.82)	73.91 (72.00–76.09)	75.00 (72.80–77.17)	71.43 (68.65–74.39)	77.27 (74.78–79.77)
Combo 3	0.81 (0.79–0.83)	86.36 (84.22–88.53)	62.50 (60.66–64.32)	86.96 (84.61–89.31)	61.29 (58.45–64.11)

*AUC* area under the receiver operating characteristic curve, *PLGC* precancerous lesions of gastric cancer, *NPLGC* Non-precancerous lesions of gastric cancer, *Combo* combination of patients for training and validation cohorts, *PPV* positive predictive value, *NPV* negative predictive value

Statistical values are presented with the 95% CIs when applicable

AUCs obtained with three different combinations of patients were statistically compared with each other in each classification and each cohort (*P < 0.05; **P < 0.01)

### Independent external validation of the AITonguequiry model

**Fig. 4:**
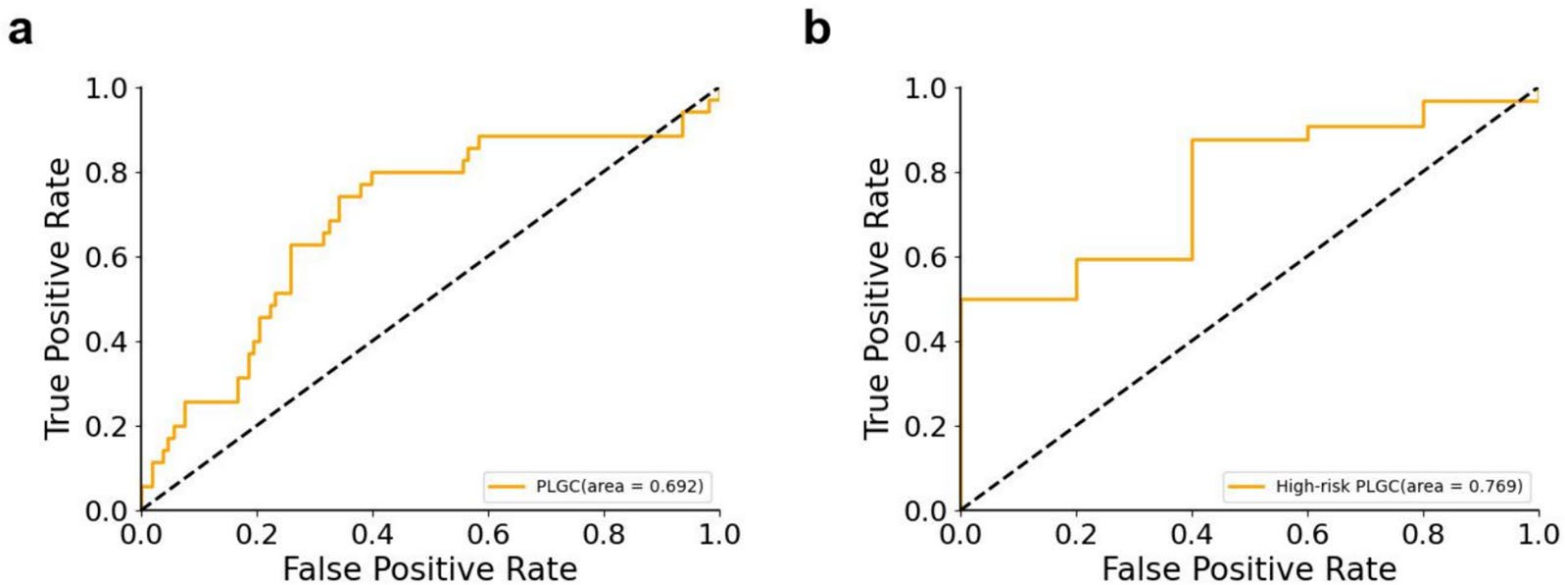
Receiver operating characteristic (ROC) curves during the external validation using Single-Tongue. Presence or absence of NPLGC/PLGC in the external validation cohorts and presence or absence of low-risk PLGC/high-risk PLGC in the external validation cohorts. *PLGC* precancerous lesions of gastric cancer, *NPLGC* non-precancerous lesions of gastric cancer

Moreover, 143 participants were recruited for independent external validation from the China-Japan Friendship Hospital. In the independent validation cohort, the AUC of PLGC was 0.69, and the AUC of high-risk PLGC was 0.77 (Fig. 4; Table 5). These results demonstrate the effectiveness of the AITonguequiry model in independent external validation. The patients in the China-Japan Friendship Hospital come from all over the country and have a diverse sample, which verifies the broad applicability of our proposed method. In the later stages, we will further incorporate more multi-center data and external validation to enhance and validate the model's generalization performance.

**Table 5:** Diagnostic performance of Single-Tongue for the assessment of NPLGC/PLGC and low-risk PLGC/high-risk PLGC in the external validation cohorts

	AUC	Specificity (%)	Sensitivity (%)	PPV (%)	NPV (%)
Step 1:					
NPLGC/PLGC	0.69 (0.66–0.72)	80.56 (79.36–81.73)	40.00 (36.36–43.56)	40.00 (35.58–44.18)	80.56 (78.61–82.52)
Step 2:					
Low-risk PLGC/high-risk PLGC	0.77 (0.74–0.79)	33.33 (26.49–40.00)	88.24 (87.61–88.90)	93.75 (92.60–94.85)	20.00 (15.19–24.81)

*AUC* area under the receiver operating characteristic curve, *PLGC* precancerous lesions of gastric cancer, *NPLGC* non-precancerous lesions of gastric cancer, *Single-Tongue* single-modality deep learning risk prediction with tongue images, *Single-Inquiry* single-modality deep learning risk prediction with inquiry information, *AITonguequiry* multimodality deep learning risk prediction with tongue images and inquiry information, *PPV* positive predictive value, *NPV* negative predictive value

## Discussion

Given the significant burden of GC in China and globally, it becomes imperative to adopt cost-effective approaches for large-scale screening of PLGC risk prediction in the natural population. While gastroscopy and pathological tests serve as the gold standard for diagnosing gastric diseases, they are not suitable for widespread application in the natural population. To address this pressing issue, there is an urgent need to develop noninvasive and effective screening and diagnostic methods to provide tailored recommendations for endoscopy. Deep neural network technology offers a promising avenue for advancing healthcare systems by providing heightened accuracy and computational power, thereby playing an increasingly vital role in disease risk prediction. In our study, a multimodality AI-assisted pre-endoscopic screening model based on tongue images and inquiry information (AITonguequiry) was constructed for the first time, adopting a hierarchical prediction strategy, achieving tailored endoscopic recommendations.

In this study, the diagnostic accuracy of AITonguequiry was significantly better than that of Single-Tongue or Single-Inquiry in assessing PLGC and high-risk PLGC. In the evaluation of PLGC, the AUC in the validation cohort was 0.74 (Fig. 2a; Table 3). Thus, AITonguequiry was effective for the assessment of PLGC. In the evaluation of high-risk PLGC, the AUC in the verification cohort was 0.82, showing that AITonguequiry was effective for the assessment of high-risk PLGC (Fig. 2b; Table 3). Our AITonguequiry has superior performance compared to existing screening methodologies, with the AUC value enhancing 45% in terms of PLGC screening (0.74 vs 0.51, p < 0.05) and 52% in terms of high-risk PLGC screening (0.82 vs 0.54, p < 0.05). The above analysis revealed that the multimodality models were effective in discriminating patients with PLGC from participants without PLGC, and could also effectively differentiate patients with high-risk PLGC from those with low-risk PLGC. Thus, the fusion model of tongue images and inquiry information further improved the diagnostic value (Fig. 2; Table 3).

The risk prediction of PLGC using tongue images and inquiry information can serve as an effective, noninvasive auxiliary diagnostic method that can support primary healthcare systems worldwide. With the recent advancements in deep neural networks, significant progress has been made in standardizing tongue images and inquiry information risk prediction, especially in TCM. Many results have been achieved in tongue image preprocessing, tongue detection, segmentation, feature extraction and tongue analysis [34]. Xu et al. developed a multi-task joint learning model to segment and classify tongue images using deep neural networks, which can optimally extract tongue image features [35]. Li et al. pioneered the use of both tongue images and the tongue-coating microbiome as diagnostic tools for gastric cancer [25]. In the study of screening precancerous lesions of gastric cancer, Ma et al. constructed the first deep learning model for screening precancerous lesions of gastric cancer based on tongue images [24]. Compared with Ma et al. [24] research on hospital cohorts, our AITonguequiry was applied to a cohort of 1035 patients in areas with a high incidence of gastric cancer. At the same time, we improved the accuracy of precancerous lesions to 73%, which is a 9% improvement. To our knowledge, AITonguequiry is the first simplified, novel, AI-based multimodal model processing tool that can accurately and noninvasively identify PLGC and high-risk PLGC. Moreover, our AITonguequiry multimodal model, for the first time, incorporates inquiry information and employs a hierarchical prediction strategy, resulting in more refined endoscopic recommendations. We advocate for patients predicted with PLGC to undergo endoscopic examination, with a concurrently recommending prompt endoscopic examination for those predicted with high-risk PLGC.

The AITonguequiry model has demonstrated an enhanced ability to detect PLGC before endoscopy. In a cross-sectional survey conducted by the Digestive Endoscopy Society of the Chinese Medical Association in 2014, involving 8892 patients diagnosed with chronic gastritis via gastroscopy across 10 cities and 33 centers, it was found that 56.7% of patients had PLGC [36, 37]. Notably, among patients recommended for endoscopic examination according to the AITonguequiry model, the detection rate of PLGC reached 73%. Consequently, our method exhibited a 28.7% increase in PLGC patient detection compared to direct endoscopic screening. With AITonguequiry, operators only need to execute the daily data acquisition workflow to automatically analyze critical information, rendering the model highly convenient for clinical applications. This enhances patient screening efficiency and reduces patient burden.

The AITonguequiry can be integrated into clinical practice through collaboration with hospitals and communities. The AITonguequiry, as presented herein, primarily focuses on early detection and personalized screening recommendations. Deployment in a clinical environment involves procedures such as data collection, integration with existing medical systems, and training of healthcare personnel. Potential technical challenges during implementation include issues such as data quality, model interpretability, and integration with electronic health records. Strategies to address these challenges will include implementing robust data preprocessing techniques, enhancing model transparency, conducting pilot studies for workflow integration, and developing educational programs for clinicians and patients. In summary, AITonguequiry demonstrates strong clinical applicability.

According to the principles of TCM, tongue diagnosis and inquiry are integral components of the four diagnostic methods in TCM. The characteristics of the tongue's shape, color, size, and coating, as detected through tongue images, can reflect an individual's health status and the severity/progression of diseases [38]. In addition, inquiry information, including demographics, behavior, and physical symptoms, plays a crucial role in understanding the disease and medical history. In this study, we discovered that useful features for predicting PLGC can be extracted from tongue images using a deep learning model. This finding indicates that the evaluation of human health based on tongue characteristics, as proposed by TCM theory, has scientific grounds. Furthermore, we found that the deep-learning model can extract informative features for PLGC prediction from inquiry information, suggesting that the evaluation of human health based on demographic, behavioral, and physical symptom information in inquiries, as guided by TCM theory, is scientifically supported. We believe that with the widespread application of AI and deep learning methods, tongue images and inquiry information can potentially become cost-effective, non-invasive and acceptable approaches for predicting and screening PLGC, which will also lead to significant socio-economic impacts.

Nevertheless, our research has some limitations. One limitation of our study is the restricted number of patients included. For future research, it will be essential to involve a larger screening population to improve the training of the deep learning model. Additionally, the interrogation data may be inconsistent and subjective, and may cause feature capture bias. The data collection process will be further improved in the following research, and more quantitative indicators will be added to assist in the diagnosis [39]. At the same time, there is potential to expand by creating a semantic dataset of tongue images and establishing a multimodal large language model for PLGC prediction, offering personalized predictions and detailed explanations for clinicians and patients. These areas merit further exploration in the future.

## Conclusions

In conclusion, this study introduces a hierarchical AI-based multimodal non-invasive method for pre-endoscopic risk screening. All these findings indicate that AITonguequiry is a non-invasive method for predicting and assessing PLGC and high-risk PLGC, showcasing its strong performance in graded screening and diagnosis. Our method has a good potential for widespread clinical use, and further studies in larger patient populations are needed. We will further promote the application of AITonguequiry in PLGC risk prediction and provide tailored recommendations for endoscopy to improve the diagnosis rate, especially high-risk PLGC, in the natural population. Moreover, this study provides scientific support for the theory of tongue images and inquiry information diagnosis in TCM.

## Data Availability

The data of individual deidentified participants will not be shared, but it is available upon request via email: shaoli@mail.tsinghua.edu.cn.

## Abbreviations

AI: Artificial Intelligence
AITonguequiry: A multimodality Artificial Intelligence-assisted pre-endoscopic screening model based on Tongue images and inquiry information
AUC: Area under the curve
CI: Confidence interval
CNNs: Convolutional neural networks
EGD: Esophagogastroduodenoscopy
GC: Gastric cancer
Hp: Helicobacter pylori
NPLGC: Non-precancerous lesions of gastric cancer
PLGC: Precancerous lesions of gastric cancer
ROC: Receiver operating characteristic
TCM: Traditional Chinese Medicine
